# Coping under pressure: police-specific stressors and mental health in Catalonia police forces

**DOI:** 10.3389/fpsyt.2026.1800257

**Published:** 2026-04-21

**Authors:** Ana F. Moreno, Patrícia Oliveira-Silva, Rowena Hill, Susanna Rubiol Vilalta

**Affiliations:** 1 Human Neurobehavioral Laboratory - HNL; Research Centre for Human Development - CEDH, Faculty of Education and Psychology, Universidade Católica Portuguesa (FEP-UCP), Porto, Portugal; 2 School of Social Sciences, Nottingham Trent University, Nottingham, United Kingdom; 3 Faculty of Psychology, Education and Sport Sciences Blanquerna - Faculty of Psychology, Education and Sport Sciences (FPCEE) Blanquerna, University Ramon Llul, Barcelona, Spain

**Keywords:** police, organizational stress, operational stress, coping, gender, years of service, wellbeing

## Abstract

**Introduction:**

Police officers are exposed to elevated psychological risks due to both operational and organizational stressors. Additionally, police officers tend to resort to avoidant coping strategies, which exacerbate poor mental health outcomes, such as burnout and PTSD.

**Methods:**

This study aims to examine clinical symptoms (stress, anxiety, depression), coping styles, and perceived stressors among police forces from Catalonia, Spain. A total of 741 officers completed an online survey comprising DASS-21, PSQ-Op, PSQ-Org, Brief COPE and brief open-ended questions.

**Results:**

Overall, both operational and organizational stressors were significant predictors of clinical symptoms, with the latter revealing a more pronounced impact. Avoidant coping emerged as the strongest risk factor for distress, while problem-focused coping emerged as a possible protective factor, especially against depression. Both gender and years of service influenced coping strategies: i) female officers reported higher use of adaptive coping, while male officers scored higher in avoidant coping; and ii) more experienced officers reported lower anxiety symptoms but also lower use of active coping strategies.

**Discussion:**

These findings underscore the importance of addressing both organizational culture and individual-level factors in promoting psychological resilience, while considering gender and career stage to support sustainable mental health within police forces.

## 1 Introduction

Policing is widely recognized to be one of the most stressful occupations, with multiple levels of relevant stressors negative and significantly affecting police officers’ (POs) physical and mental health. In comparison to the general population, POs frequent exposure to violence, traumatic incidents and public safety demands present a higher psychological risk, which commonly translates into chronic trauma, traumatic stress, post-traumatic stress disorder (PTSD), anxiety, among others (1–4). Given the inherently intertwined nature of operational and organizational stressors in policing, an integrated assessment of these stressors alongside psychosocial variables is essential. This paper will focus on exploring the organizational and operational stressors independently, coping strategies, and mental health outcomes among Spanish officers, while examining the influence of gender, years of service, police specific stress, and coping variables on clinical symptoms.

### 1.1 Stressors: operational vs organizational

In the police context, occupational stressors are commonly distinguished as operational stressors, which result from police fieldwork and job nature, and organizational stressors, arising from within the police institution structure and culture (5). Among the most well-established operational factors, is the frequent and chronic exposure to critical incidents, most of the time dangerous and emotionally intense. This cumulative exposure to violence, death, and life-threatening situations can lead to a variety of mental health issues, particularly anxiety-related symptoms, such as intrusive memories, hypervigilance, and physiological arousal, leading to increased risk of traumatic stress and compromise police wellbeing, performance efficacy and public safety (2–4, 6–9). Other consistent operational stressors highlighted by the literature have been work overload, shift work, life-or-death decision making, and the unpredictable nature of some situations. Overtime, these impact physical health, such as sleep disorders and fatigue, and mental health, often translating into chronic stress and increased cardiovascular risk (3, 10–12).

On the other hand, organizational stressors have been recognized as a key factor in understanding policing as a high-risk occupation, for both physical and mental health. These stressors derive not only from the police organizational culture itself, but also structural and interpersonal factors, such as bureaucratic and administrative demands, role ambiguity, inadequate equipment, work-life conflict, organizational pressure, poor leadership, internal conflicts, organizational climate and fairness, and perceived insufficient institutional support (3, 4, 6, 13–17). While operational stressors are a significant police-related stress source, organizational factors can play a moderating role in key variables for police performance (e.g., job satisfaction), and intensify the psychological impact of police duties, contributing to cumulative stress, burnout, and decreased wellbeing (3, 6, 15, 16, 18–20). These findings support the need to assess organizational and operational stressors separately, while recognizing their combined influence in police occupational stress.

### 1.2 Stressors and coping differences among the police

At the individual level, research has highlighted several sociodemographic and occupational differences that impact how stress is experienced, and strategies officers employ to cope with it. A recent systematic review led by Alves and colleagues (6) found that personality traits, such as neuroticism and low emotional stability, could portray a higher risk for psychological wellbeing, while resilience, emotional regulation, and hardiness served as protective factors. Coping strategies appear especially relevant, as across studies, avoidant coping (e.g., denial, distancing, substance use) has been consistently associated with increased burnout and distress, while problem-focused and active coping strategies, such as support-seeking, are generally predictive of better psychological adjustment (20–23).

Among sociodemographic variables, gender differences have been consistently reported with female POs tending to report higher levels of anxiety and depressive symptoms, often linked to internalizing stress mechanisms, while male POs are more likely to exhibit externalizing behaviours (2, 3, 11). Additionally, compared to their male counterparts, female POs tend to experience safety-related, interpersonal, and overall organizational stressors as more intense, and tend to show higher burnout scores, work-family conflict, and slower career advancement. However, they are more likely to use emotion-focused and active coping strategies, such as seeking support, and positive reappraisal, while male officers tend to resort to avoidant coping, including substance misuse (24–27). Another relevant yet controversial sociodemographic variable is age and years of service. While some studies find no consistent age and/or years of service-related differences among police officers (20, 28, 29), others suggest otherwise. Generally, less experienced officers tend to perceive higher stress levels, burnout, and worse psychological wellbeing, probably due reduced exposure to high-risk tasks, whereas senior officers may benefit from accumulated professional experience, effective coping repertoire, and build resilience (3, 26). However, prolonged service may also lead to as cumulative exposure to trauma increasing vulnerability to PTSD, emotional exhaustion, and physical health issues (1, 3, 7). Together, these results underscore the relevance of individual variable and coping mechanisms, especially considering adaptive trajectories within policing contexts.

### 1.3 The Spanish context: current research and goals

The Spanish reality mirrors the international findings, with recent research supporting higher psychosocial risk and negative impact of perceived stress and occupational factors on officers’ job satisfaction and health (8, 9, 15, 18). Moreover, a study lead by Luceño-Moreno and colleagues (15) found significant differences according to police ranks, with lower-ranking officers reporting lower perceived control and reduced institutional support and recognition. Specifically, within Catalonian police forces, some critical stressors have been highlighted such as the perceived lack of institutional support, rigid hierarchical structure, limited career progression, and challenges related to internal diversity and recognition, particularly affecting female and ethnic-minority officers (30). Nonetheless, a notable gap in the literature remains, with few studies representing regional police differences.

Therefore, we examined a sample of Catalonian Spanish police forces, with three main goals: i) to characterize main operational and organizational stressors, coping styles and clinical symptoms (depression, anxiety and stress); ii) to examine differences in occupational stressors, coping strategies and clinical symptoms according to gender and years of service; and iii) to explore the impact of gender, years of service, police stressors and coping on officers’ clinical symptoms. Thus, we hypothesize that (H1) gender-related significant differences will emerge, with females overall reporting higher levels of organizational stress, higher depressive and anxiety symptoms, and greater use of emotion-focused coping strategies, compared to higher use of avoidant coping in male officers; (H2) years of service-related significant differences concerning occupational stress and coping, with less experienced officers showing higher operational stress, but higher organizational stress in more experienced officers,; and (H3) higher operational and organizational stressors and avoidant coping will be significant predictors of worse clinical symptoms outcomes, whilst problem and emotion-focused coping have a protective role.

## 2 Methods

### 2.1 Procedure

This study is part of a broader international project (*Mental Health NeuroForce*) that focuses on stress and mental health among police forces on Portugal, United Kingdom and Spain. This cross-sectional study was carried out with Catalonian police forces, in Spain. The research adhered to deontological ethical principles and fully complied with the General Data Protection Regulation (GDPR) under the EU legal framework (31), and was approved in its main institution by the Ethics Committee for Technologies, Social Sciences and Humanities at Universidade Católica Portuguesa (CETCH-UCP).

An online survey was distributed between June and October of 2023, using a hyperlink via police unions for data collection. The procedure followed to meet both the general and specific research goals, using online Google Forms to reach the entire intended participant sample. The online survey was used to collect data, including four self-report questionnaires and brief open-ended questions. This was preceded by an informed consent form, including a brief presentation of the research, confidentiality, and other ethical requirements. The online survey was shared via the participants’ personal email addresses through police unions in Catalonia.

#### 2.1.1 Sample

The sample comprised 741 officers from Catalonian police forces (*Mossos d’Esquadra*), including several police units, particularly those involved in public safety and public order, administrative policing, judicial policing and criminal investigation, and patrolling and ensuring the safety of highways within Catalonia. No exclusion criteria were applied, with exception for technical staff (non-police officers) involved in operational support functions. The mean age of participants was 45.58 years (*SD* = 7.38) and consisted mostly of male officers (75.0%). Regarding education levels, the majority completed upper secondary education, representing 40.2% of the sample, followed by POs with an undergraduate degree (35.7%), and a minority having completed ESO (9.6%), master degree (7.8%), and other educational non-specified backgrounds (6.7%). In terms of occupational-related characteristics, participants were distributed across different police ranks, with the majority among the basic rank (86.9%), 12.2% in the intermediate rank, and a small percentage in higher ranks (0.5%). Additional sociodemographic information on the sample is represented in **Table 1**.

**Table 1:** Sociodemographic information on gender, educational level, years of service, and rank.

Variables	Categories	n	%
Gender	Male	557	75
	Female	184	24.8
	Prefer not to say	2	0.4
Educational Level	Upper Secondary Education	299	40.2
	ESO (Compulsory Secondary Education)	71	9.6
	Undergraduate Degree	265	35.7
	Master	58	7.8
	Other	50	6.7
Years of Service	1–5 years	69	9.3
	5–10 years	6	0.8
	10–15 years	114	15.3
	15–20 years	181	24.4
	20–25 years	183	24.6
	25.30 years	148	19.9
	+30 years	42	5.7
Rank	Basic rank	646	86.8
	Inspector Rank	3	0.4
	Intermediate Rank	91	12.2
	Senior Rank	1	0.1

#### 2.1.2 Instruments

The online survey compiled 4 sections:

1) Sociodemographic (age, sex, education level) and occupational information (years of service and ranking).

2) Depression, Anxiety and Stress 21 (DASS-21; 32; validated for the Spanish population by 33): 21-item scale, using a 4-Likert scale (0 - Never to 3 – Almost Always). This is a shorter version of the full DASS scale aiming to assess clinical indicators of psychological distress, divided in 7-item subscales of Depression (α=0.88), Anxiety (α=0.83), and Stress (α=0.83). Past studies have recommended a second-order factor for improved model fit and a general score of the scale, including Spanish validation studies (33–35).

3) Brief COPE (36; validated for the Spanish population by 37): 28-item scale, using a 4-Likert scale (1- I haven’t been doing this at all; 4 – I’ve doing this a lot), aims to assess coping strategies used by individuals. The Spanish validation has been focused on the 14-factor structure of 2 items each, with an overall reasonable internal consistency (α=0.75). Regarding each subscale, the Cronbach’s alpha revealed an acceptable value for all subscales considering the 2-item organization of each factor, ranging between 0.58 and 0.91 (active coping, α=0.73; planning, α=0.70; instrumental support, α=0.81; positive reframing, α=0.72; emotional support, α=0.78; acceptance, α=0.91; religion, α=0.73; venting, α=0.79; self-blame, α=0.58, humour, α=0.78; behavioural disengagement, α=0.84; denial, α=0.76; self-distraction, α=0.65; and substance use, α=0.77). Researchers favour the Brief COPE for police studies because it captures a broad profile of coping patterns among police officers (e.g., 26).

4) Police Stress Questionnaires (PSQs) – Operational Police Stress Questionnaire (PSQ-Op) and Organizational Police Stress Questionnaire (PSQ-Org) (5, 38; validated for the Spanish population by Rubiol Vilalta et al., 39): 20-item scales, using a 7-Likert scale (1- No Stress At All; 4- Moderate Stress; 7- A Lot of Stress), aiming to separately assess main job stressors derived from the police occupation (PSQ-Op) and stressors related to police organizational functioning (PSQ-Org). The validation process for Spanish population revealed a good internal consistency (PSQ-Op: α=0.96; PSQ-Org: α=0.97), which was consistent to the original study (PSQ-Op: α=0.90; PSQ-Org: α=0.89). These two instruments were specially designed for police populations, allowing for distinction of operational and organizational stressors, recognizing the equally significant role of both for police wellbeing.

### 2.2 Data analysis

Regarding data analysis, we used IBM SPSS Statistics (30.0), to conduct descriptive and multivariate analyses of the data. Missing data were handled according to the multiple imputation method which allows to maintain statistical power while minimizing the risk of bias (40). Normality of the data was assessed using skewness and Kurtosis, indicated for larger sample sets (N> 300-500), suggesting an approximate value threshold of ±1.96 for skewness and Kurtosis (41–43), while more recent studies advocate that a higher kurtosis value (≤ |4|) can still be considered acceptable for parametric tests in larger sample sets (41). All variables violated normality assumptions based on the Kolmogorov-Smirnov test (p <.05). However, an examination of skewness and kurtosis revealed a normal distribution or mild deviations (|Skewness| < 1, |Kurtosis| < 2), supporting the use of parametric tests, except for PO’s rank (Skewness = 2.25, Kurtosis = 3.11), and Brief COPE subscales of *substance use* (Skewness = 3.56, Kurtosis = 13.18), and *denial* (Skewness = 2.21, Kurtosis = 5.09).

Reliability and confirmatory factor analysis (CFA) of Brief COPE and DASS-21 was used to assess the fit of these instruments to Catalonia police sample using IBM SPSS AMOS (30.0). Regarding the PSQ-Op and PSQ-Org instruments, reliability and CFA are reported according to the validation process. T-tests, ANOVA and respective non-parametric alternatives were conducted to assess differences of clinical symptomatology, police operational and organizational stress, and coping strategies according to gender and years of service. Finally, a multivariate multiple regressions were performed to assess the effect of coping, operational and organizational stress, gender, and years of service on police officers’ clinical symptomatology.

## 3 Results

Firstly, descriptive analysis of each instrument was performed (**Table 2**). Overall participants scored within the expected of the general Spanish population average (37), except for emotional support, self-blame, denial, and behavioural disengagement, where POs scored below the expected average. Concerning depressive, anxious, and stress symptoms, Spanish POs scored above the expected for each dimension of the DASS-21 (33).

**Table 2:** Descriptive information of brief COPE, DASS-21, PSQ-Op and PSQ-Org compared to general Spanish population.

Scale	Subscales	Police samples mean (SD)	General spanish population mean (SD)
Brief COPE	Instrumental Support	2.29 (0.74)	2.94 (0.73)
	Active Coping	2.94 (0.74)	2.97 (0.55)
	Positive Reframing	2.49 (0.83)	2.98 (0.66)
	Planning	2.74 (0.76)	3.03 (0.54)
	Emotional Support	**2.20 (0.80)**	3.05 (0.72)
	Venting	1.97 (0.70)	2.24 (0.75)
	Humour	2.41 (0.89)	2.53 (0.89)
	Acceptance	2.96 (0.73)	3.10 (0.58)
	Religion	1.37 (0.66)	1.70 (0.84)
	Self-Blame	**1.96 (0.70)**	2.68 (0.99)
	Self-distraction	2.27 (0.81)	2.79 (0.63)
	Denial	**1.27 (0.51)**	2.80 (0.75)
	Substance Use	1.15 (0.48)	1.34 (0.63)
	Behavioural Disengagement	**1.40 (0.58)**	2.33 (0.67)
DASS-21	Total Distress	**35.10 (12.58)**	18.03 (12.39)
	Stress	**13.88 (4.97)**	8.06 (4.66)
	Depression	**11.05 (4.78)**	5.08 (4.75)
	Anxiety	**10.17 (3.86)**	4.89 (4.39)
PSQ	Operational (PSQ-Op)	3.31 (1.40)	–
	Organizational (PSQ-Org)	3.74 (1.56)	–

N=741; Values in bold signal above or below the expected average considering the Spanish population mean.

### 3.1 Instruments factorial analysis

To assess the fit of the validated instruments to this specific sample, we ran confirmatory factor analysis (CFA) of each instrument, following advised standard thresholds (44, 45). Firstly, concerning DASS-21 we ran two models following the discussion between the 3-factor model and the second-order 3-factor model (psychological distress). The 3-factor model shows an overall acceptable fit to the data considering large sample sizes (*χ*²(91) = 270.79, *p* <.001; CFI = .983, TLI = .960, RMSEA = .038; SRMR = .127; AIC = 592.79^1^) and acceptable model stability (Hoelter’s N(0.05) = 313). Yet, the inclusion of a general factor to the 3-factor structure shows a much-improved model fit (*χ*²(83) = 102.59, *p* = .071; CFI = .998, TLI = .995; RMSEA = .018; SRMR = .0163; AIC = 440.59; Hoelter’s N(0.05) = 760), supporting the inclusion of a general psychological distress score.

Regarding Brief COPE, the 14-factor model revealed a good fit to the data (χ²(185) = 380.73, p <.001; CFI = .972, TLI = .942; RMSEA = .038; SRMR = .056; AIC = 878.73), all indicating a well-fitting model and acceptable model stability (Hoelter’s N(0.05) = 424). Despite not being contemplated in previous Spanish validation studies, the international literature has proposed to organise these into broader coping constructs, namely three higher-order coping dimensions: emotion-focused coping (acceptance, emotional support, humour, positive reframing, religion, self-blame; venting), problem-focused coping (active coping, instrumental support, planning), and avoidant coping (behavioural disengagement, denial, self-distraction, substance use) (36, 46). A model including these higher-order coping dimensions was tested, which demonstrated very good fit to the data and sample size (χ²(12) = 24.07, p = .020; CFI = .996, TLI = .967; RMSEA = .037; SMSR = .022; AIC = 238.07) and will be considered for multivariate analysis.

Concerning PSQ-Op and PSQ-Org, following the previous validation of the questionnaires, the unidimensional models of both instruments were considered based on theoretical assumptions and parsimony indices. The unidimensional model of PSQ-Op shows an excellent fit to the Spanish Catalonian police population (*χ*²(61) = 65.56, *p* = .322; CFI = .999; TLI = .998; RMSEA = .012; SRMR = .017; AIC = 403.56), with a Cronbach’s alpha of 0.958. Similarly, the unidimensional model of PSQ-Org shows an excellent fit (*χ*²(59) = 65.65, *p* = .257; CFI = .999; TLI = .997; RMSEA = .015; SRMR = .014; AIC = 407.65), with a Cronbach’s alpha of 0.968.

### 3.2 Gender and years of service: differences on clinical symptoms, coping and police stressors

To understand associations and differences among study variables, a correlation matrix and differences tests were performed. For differences analysis purposes, and following recommendations for appropriate sample size per group, all sociodemographic categories smaller than 10 participants were excluded.

As expected, subscales from the same instrument correlated significantly. Among the variables, police operational and organizational stressors were significant and strongly correlated with each other (*r* = .81; *p* <.05), moderately to depressive, anxiety and stress symptoms (PSQ-Op: *r* = .53, *r* = .43, *r* = .40; PSQ-Org: *r* = .52, *r* = .44, *r* = .41; *p* <.05), and significant but modestly correlated with emotion-focused (PSQ-Op: *r* = .12; PSQ-Org: *r* = .11; *p* <.05) and avoidant coping (PSQ-Op: *r* = .26; PSQ-Org: *r* = .27; *p* <.05). Interestingly, none of the sociodemographic variables correlated significantly with police related stressors. Yet, problem-focused and emotion-focused coping correlated significant but modestly to sociodemographic variables, particularly female (*r* = .18; *p* <.05) and younger officers (*r* = -.13; *r* = -.18; *p* <.05), who seem to resort more often to these types of coping. Both clusters of emotion-focused (stress: r = .17; depression: r = .11; anxiety: r = .13; p <.05) and avoidant coping (stress: r = .44; depression: r = .48; anxiety: r = .46; p <.05) were significantly correlated with all three dimensions of clinical symptomatology. These are presented bellow in **Table 3**.

**Table 3:** Pearson correlation coefficients for sociodemographic variables, police-related stressors, clinical symptoms and coping.

Variables	1.	2.	3.	4.	5.	6.	7.	8.	9.	10.	11.
1. Gender	—										
2. Education	.10^**^	—									
3. Years of Service	-.13^***^	-.19^***^	—								
4. PSQ-Op	-.06	.01	.03	—							
5. PSQ-Org	-.07	.02	.06	.81^***^	—						
6. DASS-21 Stress	-.03	-.02	.02	.52^***^	.52^***^	—					
7. DASS-21 Depression	-.07^*^	-.03	.03	.43^***^	.44^***^	.79^***^	—				
8. DASS-21 Anxiety	-.05	-.02	-.03	.40^***^	.41^***^	.77^***^	.77^***^	—			
9. Problem-Focused Coping	.18^***^	.04	-.13^***^	.03	.05	.03	-.06	.00	—		
10. Emotion-Focused Coping	.18^***^	.02	-.18^**^	.12^**^	.11^**^	.17^***^	.11^**^	.13^***^	.65^***^	—	
11. Avoidant Coping	.02	-.06	.03	.26^***^	.27^***^	.44^***^	.48^***^	.46^***^	.14^***^	.39^***^	—

N=741; Two-tailed significance Pearson correlation; *p <.05; ** p <.01; ***p <.001.

#### 3.2.1 Gender

After excluding the gender category Prefer not to say (n=2), an independent samples t-test was used to test gender differences according to clinical symptomatology, coping and police operational and organizational stressors. Levene’s test for equality of variances was conducted for each subscale, and in cases where homogeneity was not met (*p* <.05) the adjusted t-test values were used. In terms of clinical symptomatology, only the depression subscale revealed significant gender differences, with male POs reporting slightly higher levels of depressive symptoms (*t*(737) = 1.89, *p* = .045, *d* = 0.16), despite the small effect size. The total score of psychological distress, *anxiety* and *stress* did not reveal statistically significant differences among genders. Regarding coping strategies, gender did reveal significant differences for both problem-focused coping (*t*(737) = -4.86; *p* <.001; *d* = .60) and emotion-focused coping (*t*(737) = -4.65; *p* <.001; *d* = .46), with females demonstrating to resort more to both coping groups with medium to large effect. As for police operational (PSQ-Op) and organizational (PSQ-Org) stress, results showed no statistically significant differences between males and females in either operational stress or organizational stress. Although not statistically significant, male participants reported slightly higher mean scores than females in both operational stress and organizational stress. More information regarding gender differences is reported in **Table 4**.

**Table 4:** Independent samples t-test and effect sizes for DASS-21 and PSQ according to gender.

Scale	Subscale/ Dimension	Male (n=557)	Female (n=182)	t (737)	d
		M (SD)	M (SD)		
DASS-21	Total Distress	35.49 (12.91)	34.04 (11.36)	1.36	0.12
	Stress	13.96 (5.11)	13.72 (4.48)	0.56	0.05
	Depression	11.25 (4.91)	10.48 (4.32)	1.89*	0.16
	Anxiety	10.29 (3.92)	9.84 (3.64)	1.36	0.12
Brief COPE	Problem-Focused	2.59 (0.60)	2.84 (0.59)	-4.86***	0.60
	Emotion-Focused	2.15 (0.45)	2.33 (0.47)	-4.65***	0.46
	Avoidant	1.53 (0.42)	1.51 (0.42)	0.48	0.40
Police Stress	Operational Stress	3.36 (1.38)	3.16 (1.45)	1.65	0.14
	Organizational Stress	3.80 (1.55)	3.58 (1.56)	1.62	0.14

N=739; *p <.05; ** p <.01; ***p <.001; Cohen’s d values indicate effect sizes; PSQ – Police Stress Questionnaire.

**Figure 1:**
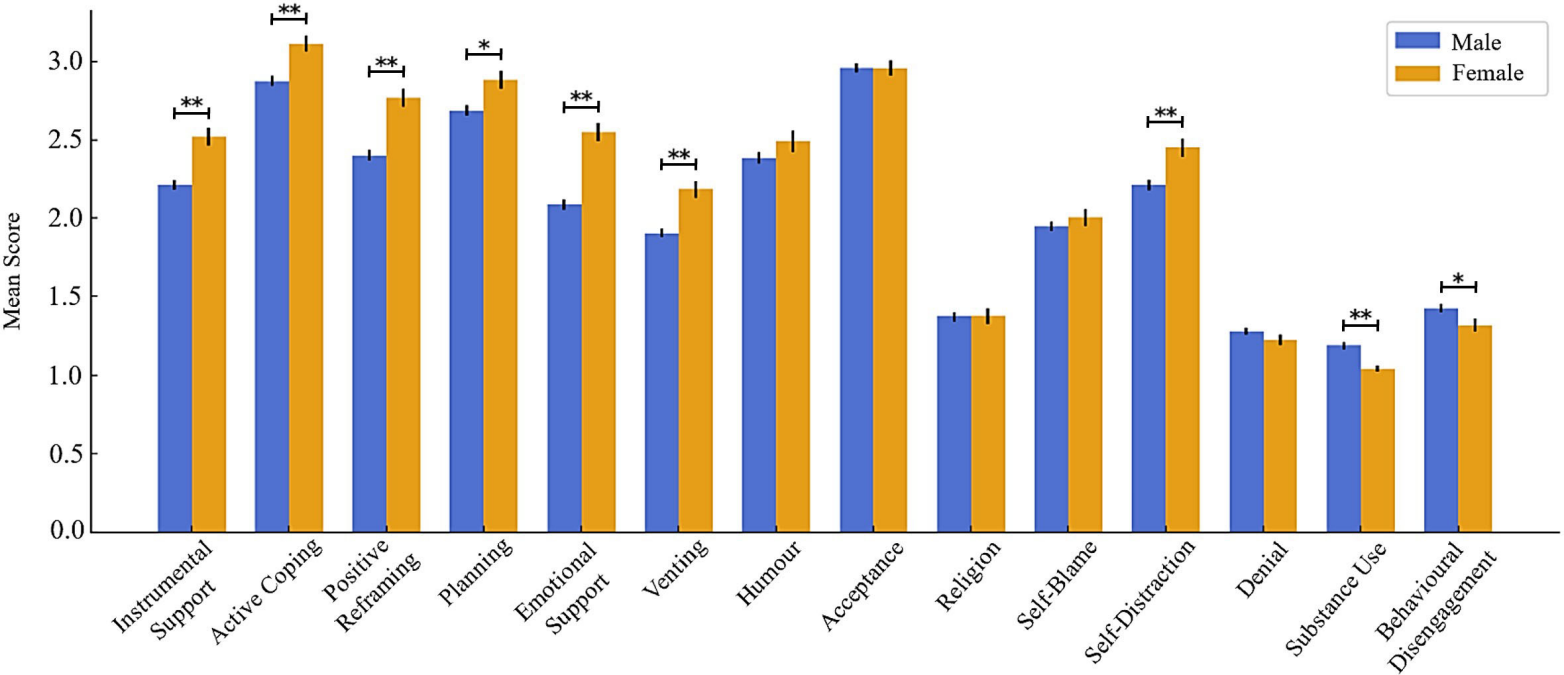
Gender differences according to coping strategies.*p <.05; ** p <.01.

Among coping strategies, further test differences were undertaken to determine gender distinctions among each individual coping strategy as illustrated on **Figure 1**. Given non-normality of substance use and denial subscales of Brief COPE, a non-parametric option was more suited for these cases (Mann-Whitney U test). Results indicated significant differences in several coping strategies, namely female POs scored significantly higher than their counterparts in instrumental support (*t*(737) = -4.96, *p* <.001, *d* = 0.40), active coping (*t*(737) = -3.80, *p* <.001, *d* = 0.33), emotional support (*t*(737) = -7.04, *p* <.001, *d* = 0.55), venting (*t*(737) = -4.74, *p* <.001, *d* = 0.38), self-distraction (*t*(737) = -3.47, *p* <.001, *d* = 0.30) and planning (*t*(737) = -3.05, *p* <.01, *d* = 0.75). Conversely, male POs only scored significantly higher on behavioural disengagement (*t*(737) = 2.04, *p* = .027, *d* = 0.21), and substance use (*U* = 45 099.50, *Z* = -4.01, *p* <.001), both avoidant coping strategies. Regarding effect sizes, the largest gender differences were observed in emotional support, positive reframing, and *self-distraction* subscales. Other coping strategies did not reveal significant differences (*p* >.05). Complete analysis can be consulted on **Appendix 1**.

#### 3.2.2 Years of service

Regarding years of service, one-way ANOVAs were performed considering differences among groups of different years of service according to coping strategies used, clinical symptoms of anxiety, depression and stress, and police operational and organizational stressors (**Table 5**). Years of service were collected in 5-year intervals. Due to unrepresentative sample size (*n* = 6), the group from 5 to 10 years was excluded from inferential analysis, following good statistical practice and avoid unreliable estimates. However, it was retained for descriptive reporting and included in **Table 5** and **Appendix 2**. When significant differences were found, *post-hoc* comparisons were conducted using Tukey’s test or Games-Howell in cases of unequal variances. Concerning clinical symptomatology (depression, anxiety and stress) and operational and organizational stress, no significant differences were found between different groups of years of service (*p* >.05). As for coping, despite not showing significant differences for avoidant coping (*p* >.05), years of service indicated a significant coping differences regarding problem-focused (*F*(5, 729) = 2.93, *p* = .013), specially higher between officers with 1–5 years (*M* = 2.41) compared to those with 20-25 (*M* = 2.21, *p* = .009) and 25–30 years of service (*M* = 2.09, *p* = .012). Emotion-focused coping also showed significant differences across different ranges, particularly higher in officers with 1–5 years of service (*M* = 2.88) compared to all other groups except for 10–15 years of service (15-20: *M* = 2.67, *p* = .004; 20-25: M = 2.59, *p* = .029; 25-30: *M* = 2.67, *p* <.001; +30 *M* = 2.65, *p* = .029). Although the subgroup with 5–10 years of service was underrepresented for inferential analyses, it showed the highest mean levels of total distress, stress, and anxiety symptoms, as well as organizational stress. They also reported greater use of problem-focused and avoidant coping clusters, and lower use of emotion-focused coping, compared with the other subgroups. Despite its limited representativeness, these findings suggest that officers in this career stage may be particularly vulnerable to emotional distress and limited effective emotional integration.

**Table 5:** One-way ANOVA and effect sizes for DASS-21 and PSQs according to years of service.

Scale/Subscales	1–5 years (n = 68)	5–10 years (n = 6)	10–15 years (n = 113)	15–20 years (n = 181)	20–25 years (n = 183)	25–30 years (n = 148)	+30 years (n = 42)	F (5, 729)	η²
DASS-21 Total Distress	23.74 (12.19)	37.17 (8.86)	35.58 (12.23)	36.72 (13.22)	34.62 (12.00)	34.27 (12.42)	35.69 (14.01)	1.31	.02
Stress	12.84 (5.31)	14.83 (4.17)	14.04 (4.55)	14.51 (5.09)	13.91 (4.88)	13.56 (5.08)	13.88 (4.87)	1.16	.02
Depression	10.09 (4.26)	11.00 (3.35)	11.04 (4.88)	11.69 (5.18)	10.73 (4.38)	11.04 (4.66)	11.36 (5.50)	1.39	.02
Anxiety	9.81 (3.58)	11.33 (3.01)	10.50 (3.99)	10.62 (3.93)	9.98 (3.63)	9.67 (3.77)	10.45 (4.71)	1.42	.02
Brief COPE Problem- Focused	2.41 (0.46)	3.14 (0.44)	2.25 (0.48)	2.17 (0.42)	2.21 (0.44)	2.09 (0.50)	2.10 (0.54)	5.40***	.03
Emotion-Focused	2.88 (0.60)	2.44 (0.48)	2.67 (0.59)	2.67 (0.59)	2.59 (0.59)	2.58 (0.63)	2.65 (0.61)	2.93*	.20
Avoidant	1.47 (0.39)	1.65 (0.34)	1.53 (0.46)	1.53 (0.40)	1.55 (0.38)	1.50 (0.38)	1.58 (0.55)	0.75	0.01
Police Stress Operational Stress	3.01 (1.30)	3.38 (1.37)	3.35 (1.49)	3.43 (1.37)	3.30 (1.38)	3.24 (1.41)	3.40 (1.54)	1.03	.007
Organizational Stress	3.45 (1.45)	4.44 (1.46)	3.61 (1.62)	3.78 (1.57)	3.85 (1.57)	3.65 (1.50)	4.13 (1.64)	1.49	.003

N=735; *p <.05; ***p <.001; η² values indicate effect sizes. The subgroup of 5–10 years of service was not considered for inferential analysis.

**Figure 2:**
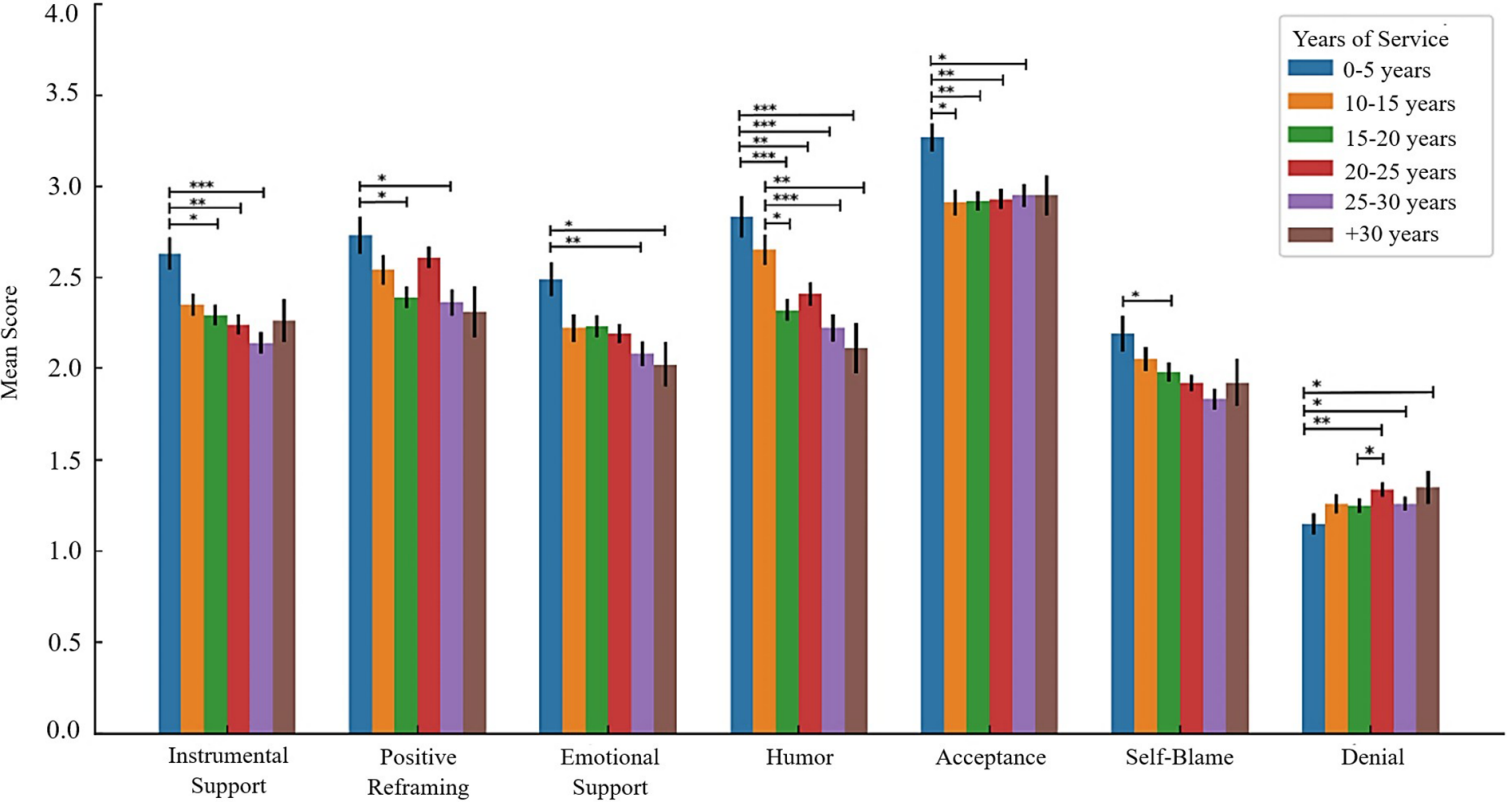
Police years of service differences according to significant coping strategies. *p <.05; ** *p* <.01; ***p <.001.

Regarding individual coping strategies, the results revealed a significant effect of years of service on instrumental support (*F*(5, 729) = 4.504, *p* <.001, η² = .030), positive reframing (*F*(5, 729) = 3.583, *p* = .003, η² = .024), emotional support (*F*(5, 729) = 3.042, *p* = .010, η² = .020), humour (*F*(5, 729) = 7.842, *p* <.001, η² = .051), acceptance (*F*(5, 729) = 2.846, *p* = .015, η² = .019), and self-blame (*F*(5, 729) = 3.108, *p* = .009, η² = .021). Overall, younger officers reported to resort more to diverse coping strategies compared to older officers, namely considered adaptive strategies such as instrumental and emotional support, and positive reframing. Interestingly, self-blame but also humour revealed a declining trend with age. Contrariwise, denial was higher among older officers compared to those in the beginning of their career, while acceptance seemed to tendentially increase with years of experience. More detailed information on *post-hoc* comparisons is provided on **Appendix 2**. No significant differences were found for other coping strategies (*p* >.05). Significant differences in coping across different years of service are represented in **Figure 2**.

### 3.3 Multivariate analysis

A series of multiple regressions were conducted to examine whether gender, years of service, coping styles, and police-related stressors predicted clinical symptoms. Underrepresented categories (*n* < 10) were excluded from the multivariate analysis to ensure model stability and interpretability. The models for stress, depression and anxiety were statistically significant, and no multicollinearity issues were detected (Variance Inflation Factor, VIF ≤ 3).

Models proved to be moderately strong (**Table 6**), with the model of stress (*F*(7, 725) = 67.78, *p* <.001) explaining approximately 40% of the variance in clinical stress levels (R² = .396). Avoidant coping was the strongest predictor of clinical stress (β = .41; *p* <.001), together with operational (β = .25; *p* <.001) and organizational stress (β = .22; *p* <.001). As for the model of depression (*F*(7, 725) = 60.30, *p* <.001), it explained approximately 37% of the variance and the same trend was observed with avoidant coping as the most relevant predictor (β = 4.13, *p* <.001), followed by organizational (β = .22; *p* <.001) and operational stress (β = .13; *p* = .010) as significant predictors. Interestingly, problem-focused coping was significant and negatively associated (β = -.12; *p* = .003), which means it might serve as an important protective factor for depressive symptoms. Finally, the model of anxiety (*F*(7, 725) = 48.88, *p* <.001) explained 32% of the variance, and similarly, avoidant coping (β = .407; *p* <.001), organizational stress (β = .195; *p* <.001) and operational stress (β = .123, *p* = .020) were strongest predictors of anxiety. Inversely, years of service showed a negative and significant predictive value (β = -.072, *p* = .022), which indicates that anxiety progressively decreases with time of experience.

**Table 6:** Multiple regression analyses with clinical symptoms as dependent variables.

Variables	Categories	DASS-21 Stress		DASS-21 Depression		DASS-21 Anxiety	
		*B* (*SE*)	95% CI	*β* (*SE*)	95% CI	*β* (*SE*)	95% CI
SD	*Gender*	.14 (.34)	[-.54,.81]	-.25 (.34)	[-.91,.42]	-.14 (.28)	[-.69,.42]
	*Years of Service*	-.06 (.10)	[-.24,.13]	-.07 (.09)	[-.25,.12]	-.18^*^ (.08)	[-.33, -.03]
Police Stress	*Operational Stress*	.90^***^ (.18)	[.55, 1.24]	.45^*^ (.18)	[.11,.79]	.34^*^ (.15)	[.05,.63]
	*Organizational Stress*	.69^***^ (.16)	[.37, 1.00]	.68^***^ (.16)	[.37,.99]	.48^***^ (.13)	[.23,.74]
Coping	*Problem-Focused*	-.44 (.32)	[-1.06,.18]	-.92^**^ (.31)	[-1.53, -.31]	-.31 (.26)	[-.82,.20]
	*Emotion-Focused*	.36 (.44)	[-.51, 1.23]	-.03 (.44)	[-.89,.83]	-.26 (.37)	[-.98,.46]
	*Avoidant*	4.02^***^ (.41)	[3.21, 4.82]	4.96^***^ (.39)	[4.17, 5.75]	3.94^***^ (.34)	[3.28, 4.61]
Model Statistics	*Intercept*	2.70^*^		2.59^*^		3.59^***^	
	R^2^	.40		.37		.32	
	*F*	67.78^***^		60.30^***^		48.88^***^	

*p < 0.05; **p < 0.01; ***p < 0.001; d*f_1_* = 7/d*f_2_* = 725; SE, standard error; SE, Standard Error; SD, Sociodemographic; All reported estimates were rounded to two decimal places.

## 4 Discussion

The current study aimed to contribute to the growing literature on police-specific stressors and mental health, while exploring main coping styles used by police officers according to gender and years of service. Among the many demands faced by police officers, organizational stressors seem to aggravate the negative impact of operational demands inherent to police work, insidiously undermining police officers’ mental health. In this context, the present study sought to disentangle the specific contributions of organizational and operational stress to police mental health outcomes, examining how different coping strategies may buffer or exacerbate these effects across dimensions of stress, depression, and anxiety, as well as differences in coping according to known risk factors as gender and years of experience.

Overall, organizational stress scores were higher than operational stress and emerged as a stronger predictor of clinical symptoms. Although operational stress also significantly predicted stress and anxiety, these findings align with the evidence that organizational dynamics play a more pervasive role in shaping psychological wellbeing and resilience in police officers (3, 14, 16, 17, 19, 38). Occupational stressors were significant predictors clinical symptoms and showed relevant correlation with avoidant coping mechanisms, while sociodemographic variables, such as gender and years of service appeared to differ considering problem-focused coping and emotion-focused coping, the latter specifically on gender. This is indicative that police occupational demands and how officers perceive and experience their work environment may shape maladaptive coping tendencies, compared to individual variables. These findings echo prior literature (13, 14, 16, 27), suggesting that organizational stressors may not only persist over time but also exacerbate the inherent emotional demands of policing.

Concerning the predictors of mental health clinical outcomes, our results showed higher prevalence of clinical symptoms in comparison to the general Spanish population, with avoidant coping emerging consistently as the strongest predictor of clinical symptoms consistent with our initial hypothesis. Contrarily, problem-focused coping stood out as a significant protective factor against depression. This supports previous research stating that resorting to adaptive coping strategies, namely active problem-solving and seeking support, may mitigate psychological strain and improve psychological resilience in high-stress occupations such as policing (2, 25, 47). Additionally, prior studies have similarly highlighted the need for training emotion and stress regulation and communication skills, due to their potential impact in buffering occupational-derived stress (13, 16, 26), and depressive symptoms (48), although they are often underrepresented in police training curricula. As hypothesized, coping styles varied across different career stages, showing an inverse relationship was observed between years of service and the use of both emotion- and problem-focused coping. While at first this could raise concerns, possibly indicating reduced coping capacity or cognitive flexibility, it may also reflect a resilience effect discussed in previous studies, meaning that more experienced officers tend to rely on fewer but more effective strategies, developed through practical experience (3, 26). However, the literature has not been consensual on this issue, often stressing the consequences of accumulated stress and exposure to traumatic events (3, 17, 26, 28), highlighting the complexity of the relationship between years of service, coping and wellbeing.

In addition, clinical symptomatology revealed expressive gender differences, with depressive symptoms particularly higher among male officers. This seems controversial with prior research and our initial hypothesis, with female POs often reporting greater emotional strain and internalizing symptoms (2, 3, 11, 25, 26). However, female officers are also more prone to resort to active coping and emotion-focused strategies, while male officers tend to rely more on avoidant coping (24–26), as was the case in the present study. Thus, it seems coping may be a key aspect to overall psychological wellbeing, as male officers scored higher on behavioural disengagement and substance use, which has been linked in previous literature to worse physical and mental health outcomes (2, 25, 27, 47). The police culture is often associated with bravery, self-reliance, and mental health stigma, which may play a relevant role specifically in a male-centred culture that may reinforce these patterns (3, 13, 27), and could help contextualize male POs’ outcomes. These findings suggest that male and female officers may perceive and experience specific occupational stressors differently, emphasizing once again the potential of coping interventions with the police (48), allied with gender-sensitive organizational policies to counteract gender-based barriers in a traditional male-centred police culture.

### 4.1 Context-sensitive recommendations

In the specific context of Spanish police and the Catalonian region these findings gain urgency. As highlighted by some recent studies and human rights organizations police forces continue to face organizational rigidity, limited resources, and perceived insufficient institutional support, with alarming prevalence of suicide and the insufficient mental health policies available (9, 30, 49, 50). In conclusion, these findings underscore the mental health risks inherent in policing, that if unaddressed can contribute to long-term negative impact on performance, increased absenteeism and burnout, and wider societal and institutional costs. This highlights the importance of integrating both operational and organizational dimensions when addressing mental health in policing and reinforcing police as at-risk occupation for mental health issues. Moreover, our findings corroborate previous findings on higher psychosocial strain among younger and female officers, emphasising the need of developing targeted interventions within police institutions that, not only address systemic issues embedded in police culture and organizational structure, but also account for individual-level differences. Intervention strategies should simultaneously foster individual resilience and address structural and cultural contributors to chronic stress, by promoting adaptive coping, emotional competence training, and leadership practices rooted in institutional recognition and fairness. Additionally, the implementation of validated screening tools such as the PSQ-Op and PSQ-Org could facilitate early identification of stress levels and allow for tailored, evidence-based interventions. In parallel, integrating confidential pathways for substance-use support may be particularly relevant to reduce risk and strengthen early professional psychological help-seeking among officers.

Future longitudinal and intervention-based research is needed to explore stress, coping and mental health variables trajectories across career stages, accounting for different sociodemographic characteristics, and evaluate the effectiveness of targeted psychosocial and organizational intervention programs. Expanding this research across diverse units and accounting for multicultural-ethnic settings would also enrich our understanding of wellbeing within the Catalonian, Spanish and international police forces. Finally, this study highlights the need for institutional investment in psychological support, socioemotional skills training, and leadership development could not only reduce chronic stress but also foster healthier, more sustainable public safety services. Future policies and interventions should prioritize mental health as a core component critical to POs’ wellbeing, and as an essential measure to sustain effective, ethical, and trusted police forces.

### 4.2 Limitations

This study provides novel insight on mental health, stressors and coping of Catalonian POs, but it is not without limitations. Firstly, the cross-sectional design limits causal interpretations about the relationships between the variables of interest. Secondly, some scales showed lower internal consistency, especially in coping subdimensions, needing further instrument refinement in future studies with police populations. Thirdly, despite no exclusion criteria being applied concerning age, there was an underrepresentation of higher-ranking officers, which limited our analysis regarding ranking as a moderating variable for police stress, coping and mental health outcomes. This extended to the specific subgroup of officers with 5 to 10 years of service, who showed higher vulnerability for emotional and organizational distress, and high reliance in avoidant coping. This highlights the need of addressing earlier-career personnel in future studies and use targeted recruitment strategies to better capture the experiences, stressors, and support needs of younger or less experienced officers, Female officers were also underrepresented, reflecting gender-imbalances typical of police culture. Additionally, we did not assess lifestyle and health-related factors, such as, working hours, sleep duration, or chronic conditions, which could present a relevant moderating role for psychological wellbeing in police personnel. Despite these limitations, the study offers several strengths that provide a strong foundation for future research. It included a large and diverse sample from a specific yet understudied police force, relevant considering its socio-political-historical background. The use of mixed-methods enriched the findings, integrating statistical models with qualitative data that offered deeper insights into officers’ experiences and perceived needs. Additionally, the inclusion of both clinical symptoms and police-specific stressors, along with coping strategies, reflect a more holistic and integrated understanding of occupational health in policing.

## 5 Conclusion

This study offers a context-sensitive foundational study on occupational stressors, coping and mental health for the Catalonian police context, a distinct regional police force with a specific institutional and socio-political context. Overall, our findings align with prior research stressing the key role of occupational stressors, particularly organizational factors, on officers’ wellbeing. The gender and career stage patterns also highlighted the underrepresentation of female and younger officers, stressing the need for future studies to adopt intentional recruitment strategies to target these clusters, subsequently enabling tailored interventions to each group’s characteristics and needs. At the same time male officers presented higher psychological vulnerability, supporting the need for organizational reforms that strengthen support and reduce mental health stigma. Reflections regarding the future include expanding across diverse police units and identities (e.g., gender, diversity, ethnic-minority status) and evaluate multilevel interventions sustaining organizational change.

## Data Availability

The database generated with the results reported in this study is not publicly available due to privacy concerns and for the protection of the officers involved. Requests to access the datasets should be directed to amoreno@ucp.pt, susanarv0@blanquerna.url.edu.

## Footnotes

1 *Note:* CFI, Comparative Fit Index; TLI, Tucker–Lewis index; RMSEA, Root Mean Square Error of Approximation; SRMR, Standardized Root Mean Square Residual; AIC = Akaike Information Criterion
