# Supplementary material for: Coping under pressure: police-specific stressors and mental health in Catalonia police forces
Source: Front Psychiatry. 2026 Apr 21;17:1800257. doi: 10.3389/fpsyt.2026.1800257 (PMC13139197; doi:10.3389/fpsyt.2026.1800257)
Supplement: Supplementary file 1 [file DataSheet1.pdf]

Appendix 1. Independent samples t-test and effect sizes for Brief COPE subscales according to Gender

**Subscale**	**Male (n=557)**	**Female (n=182)**	***t* (737)**		***d***
	**M (*SD*)**	**M (*SD*)**			
Instrumental Support	2.21 (0.72)	2.52 (0.74)	-4.96***		0.73
Active Coping	2.88 (0.75)	3.12 (0.70)	-3.80***		0.74
Positive Reframing	2.40 (0.83)	2.77 (0.78)	-5.25***		0.82
Planning	2.69 (0.76)	2.88 (0.74)	-3.05**		0.75
Emotional Support	2.09 (0.76)	2.55 (0.81)	-7.03***		0.77
Venting	1.90 (0.67)	2.18 (0.74)	-4.73***		0.69
Humor	2.39 (0.88)	2.49 (0.91)	-1.37		0.89
Acceptance	2.96 (0.74)	2.96 (0.69)	0.04		0.73
Religion	1.37 (0.67)	1.38 (0.63)	-0.08		0.66
Self-Blame	1.95 (0.69)	2.01 (0.72)	-0.98		0.70
Self-Distraction	2.21 (0.82)	2.45 (0.78)	-3.47***		0.81
Behavioral Disengagement	1.42 (0.61)	1.32 (0.51)	2.04*		0.58
			**U**	**Z**	***r***
Denial	1.28 (0.53)	1.23 (0.46)	48 458.00	-1.13	0.04
Substance Use	1.19 (0.53)	1.04 (0.25)	45 099.50***	-4.01	0.15

N=739; **p* < .05; ** *p* < .01; ***** *p* < .01; Cohen's *d* and Rank-Biserial Correlation (*r*) indicate effect sizes.
