# Supplementary material for: Coping under pressure: police-specific stressors and mental health in Catalonia police forces
Source: Front Psychiatry. 2026 Apr 21;17:1800257. doi: 10.3389/fpsyt.2026.1800257 (PMC13139197; doi:10.3389/fpsyt.2026.1800257)
Supplement: Supplementary file 2 [file DataSheet2.pdf]

Appendix 2. One-way ANOVA and effect sizes for Brief-COPE subscales according to years of service

Subscale Measure	1-5 years (*n*=68)	5-10 years (*n*=6)	10-15 years (*n*=113)	15-20 years (*n*=181)	20-25 years (*n*=183)	25-30 years (*n*=148)	+30 years (*n*=42)	*F* (5,729)	η²
Instrumental Support	2.63 (0.73)	2.91 (0.38)	2.35 (0.66)	2.29 (0.77)	2.24 (0.74)	2.14 (0.73)	2.26 (0.77)	4.50***	.030
Active Coping	3.18 (0.73)	3.33 (0.52)	2.90 (0.75)	2.98 (0.71)	2.89 (0.73)	2.86 (0.78)	2.87 (0.81)	2.05	.014
Positive Reframing	2.73 (0.84)	3.25 (0.76)	2.54 (0.85)	2.39 (0.79)	2.61 (0.78)	2.36 (0.88)	2.31 (0.90)	3.58**	.024
Planning	2.85 (0.81)	3.17 (0.52)	2.76 (0.76)	2.75 (0.70)	2.63 (0.76)	2.74 (0.81)	2.82 (0.71)	1.08	.007
Emotional Support	2.49 (0.77)	2.42 (0.97)	2.22 (0.81)	2.23 (0.81)	2.19 (0.73)	2.08 (0.75)	2.02 (0.81)	3.04**	.020
Venting	2.09 (0.66)	1.92 (0.38)	2.12 (0.73)	1.96 (0.69)	1.98 (0.69)	1.88 (0.74)	1.82 (0.67)	2.21	.015
Humor	2.83 (0.94)	2.83 (0.75)	2.65 (0.89)	2.32 (0.80)	2.41 (0.86)	2.22 (0.91)	2.11 (0.89)	7.84***	.051
Acceptance	3.27 (0.64)	3.25 (0.61)	2.91 (0.75)	2.92 (0.70)	2.93 (0.72)	2.95 (0.72)	2.95 (0.72)	2.85*	.019
Religion	1.29 (0.54)	1.42 (0.66)	1.29 (0.57)	1.41 (0.66)	1.45 (0.71)	1.31 (0.61)	1.57 (0.98)	2.14	.014
Self-Blame	2.19 (0.81)	2.00 (0.71)	2.05 (0.71)	1.98 (0.69)	1.92 (0.60)	1.83 (0.70)	1.92 (0.83)	3.11**	.021
Self-Distraction	2.38 (0.96)	2.83 (1.03)	2.25 (0.82)	2.30 (0.81)	2.30 (0.76)	2.15 (0.77)	2.26 (0.91)	1.00	.007
Behavioral Disengagement	1.27 (0.49)	1.33 (0.52)	1.42 (0.59)	1.38 (0.58)	1.43 (0.55)	1.55 (0.77)	1.55 (0.77)	1.32	.009
								**H(3)**	**η²**
Denial	1.15 (0.48)	1.42 (0.58)	1.26 (0.56)	1.25 (0.54)	1.34 (0.53)	1.26 (0.46)	1.35 (0.58)	11.35*	.015
Substance Use	1.05 (0.30)	1.00 (0.00)	1.17 (0.53)	1.19 (0.51)	1.14 (0.46)	1.17 (0.54)	1.17 (0.54)	6.79	.009

N=735; η² values indicate effect sizes. *$p < .05$; **$p < .01$; ***$p < .001$. The subgroup of 5-10 years of service was not considered for inferential analysis.
